# Structure-Function Agreement Is Better Than Commonly Thought in Eyes With Early Glaucoma

**DOI:** 10.1167/iovs.19-27920

**Published:** 2019-10

**Authors:** Donald C. Hood, Emmanouil Tsamis, Nikhil K. Bommakanti, Devon B. Joiner, Lama A. Al-Aswad, Dana M. Blumberg, George A. Cioffi, Jeffrey M. Liebmann, Carlos G. De Moraes

**Affiliations:** 1 Department of Psychology, Columbia University, New York, New York, United States; 2 Department of Ophthalmology, Columbia University, New York, New York, United States

**Keywords:** glaucoma, optical coherence tomography, perimetry, visual fields

## Abstract

**Purpose:**

To assess the agreement between structural (optical coherence tomography [OCT]) and functional (visual field [VF]) glaucomatous damage with an automated method and deviation/probability maps, and to compare this method to a metric method.

**Methods:**

Wide-field spectral-domain OCT scans, including the disc and macula, and 24-2 and 10-2 VFs were obtained from 45 healthy control (H) eyes/individuals, and 53 eyes/patients with 24-2 mean deviation (MD) better than −6 dB diagnosed as “definite glaucoma” (DG) by experts. Abnormal structure–abnormal function (aS-aF) agreement was assessed with an automated topographic (T) method based upon VF pattern deviation and OCT probability maps. Results were compared to a metric (M) method optimized for accuracy, (abnormal 24-2 glaucoma hemifield test [GHT] or pattern standard deviation [PSD], or 10-2 PSD AND abnormal OCT [quadrant]).

**Results:**

For the T-method, 47 (88.7%) of the 53 DG eyes showed aS-aF agreement, compared to 2 (4.5%) of the 45 H eyes. The aS-aF agreement for these two H eyes was easily identified as mistaken, and did not replicate on a subsequent test. Without the 10-2, the aS-aF agreement decreased from 47 to 34 (64.2%) of 53 DG eyes. For the M-method, 37 (69.8%) of the 53 DG eyes showed aS-aF agreement, while omitting the 10-2 VF resulted in agreement in only 33 (62.3%) eyes.

**Conclusions:**

There is good agreement between structural and functional damage, even in eyes with confirmed early glaucomatous damage, if both 24-2 and 10-2 VFs are obtained, and abnormal locations on the VFs are compared to abnormal regions seen on OCT macular and disc scans. This can be done in an objective, automated fashion. (ClinicalTrials.gov number, NCT02547740.)

As there is no litmus test for glaucoma, diagnosis is typically based upon characteristic changes in structural and functional tests. Since the advent of standard automated perimetry, the visual field (VF) test, typically with a 6° grid (e.g., 24-2 or 30-2 pattern), has been the functional test most commonly used, while before the advent of optical coherence tomography (OCT), fundus photographs and fundus exams served as the structural test. Today OCT tests augment, and in some clinics, replace fundus photographs. In any case, while it is generally agreed that VF and OCT information should be considered in diagnosing and staging glaucoma, there is no generally accepted method for comparing the two.

It is often said that structural damage occurs before functional (VF) damage, based largely on previous histologic post mortem studies,^1^–^4^ although these data are open to other interpretations.^5^,^6^ In any case, if structural damage does precede functional damage, then a clinical comparison between a VF (functional) test and an OCT (structural) test may be of questionable value, at least in the case of early damage. However, we^5^,^7^ and others^6^,^8^ have argued that the extent to which structural and functional glaucomatous damage agree depends upon various factors, such as the particular test and test measures used, and the baseline conditions for a particular patient. In addition, structural damage, as measured by OCT retinal nerve fiber layer (RNFL) thickness, and functional damage, as measured with VFs, parallel each other, often in a linear fashion.^5^,^9^ This suggested to us that a topographic comparison of local VF and OCT damage might show good agreement. In fact, a pilot study^7^ has suggested that a topographic comparison of abnormal regions of OCT and VFs show good agreement, if both 24-2 and 10-2 VFs tests are performed, and OCT retinal ganglion cell (RGC) and RNFL thickness maps are included. However, in that study, the analysis was post hoc; selection bias could not be ruled out; and, most importantly, the method of comparison was not objective and automated.

Here we used a prospective database of eyes classified as “definite glaucoma” (DG) by glaucoma specialists, as well as eyes of healthy controls. The DG eyes all had a 24-2 mean deviation (MD) better than –6 dB, and thus would be typically classified as early glaucoma. An automated topographic (T) method, recently described (Tsamis et al., manuscript submitted, 2019), was used to compare abnormal locations on the 10-2 and 24-2 VFs to abnormal regions on RGC and RNFL probability maps. The results for this T-method were compared to a summary metric (M) method, both optimized on the same dataset.

## Methods

### Participants

As part of Columbia University's Macular Damage in Early Glaucoma and Progression (ClinicalTrials.gov Identifier: NCT02547740), 135 individuals were recruited who had one eye with a 24-2 MD better than −6 dB. This eye was either ocular hypertensive (at least one Goldmann tonometry IOP measurement ≥22 mm Hg, normal 24-2 VFs, normal optic disc on fundus photography); glaucomatous optic neuropathy (GON) suspect (IOP measurement ≤22 mm Hg, normal 24-2 VFs, suspicious optic disc on fundus photography); or “established” glaucoma based upon the clinician's reading of functional (i.e., 24-2 and 10-2 VFs) and structural (i.e., fundus photographs and OCT) information, irrespective of IOP. The definition of normal/abnormal VF or disc photos did not follow a specific set of rules to mitigate the impact of such rules in the classification systems. Instead, definitions were left up to glaucoma experts' discretion and the final diagnosis given to patients. All participants had best corrected visual acuity ≥20/40 and open angles.

Of the 135 eyes meeting inclusion criteria, 53 (mean age, 66.93 ± 9.54 years) were categorized as DG by the referring physician. Another 37 were categorized as either probably glaucoma (PG, *n* = 17) or not glaucoma (*n* = 20). An additional 45 healthy (H) controls, with normal fundus exams and IOP ≤22 mm Hg, were recruited from hospital staff (mean age, 35.7 ± 15.2 years).

All eyes had 24-2 and 10-2 VF tests with SITA-standard protocol (Carl Zeiss Meditec, Inc., Dublin, CA, USA) and OCT scans described below. The OCT scans and 24-2 and 10-2 VF tests were completed within 4 weeks in all but one eye. In one eye, the 10-2 VF test was obtained 40 days after the 24-2 and OCT tests.

This prospective study was approved by the Institutional Review Board of Columbia University Irving Medical Center and adhered to the tenets of the Declaration of Helsinki and Health Insurance Portability and Accountability Act. Written informed consent was obtained from all participants involved in this study.

### OCT Scans

**Figure 1:**
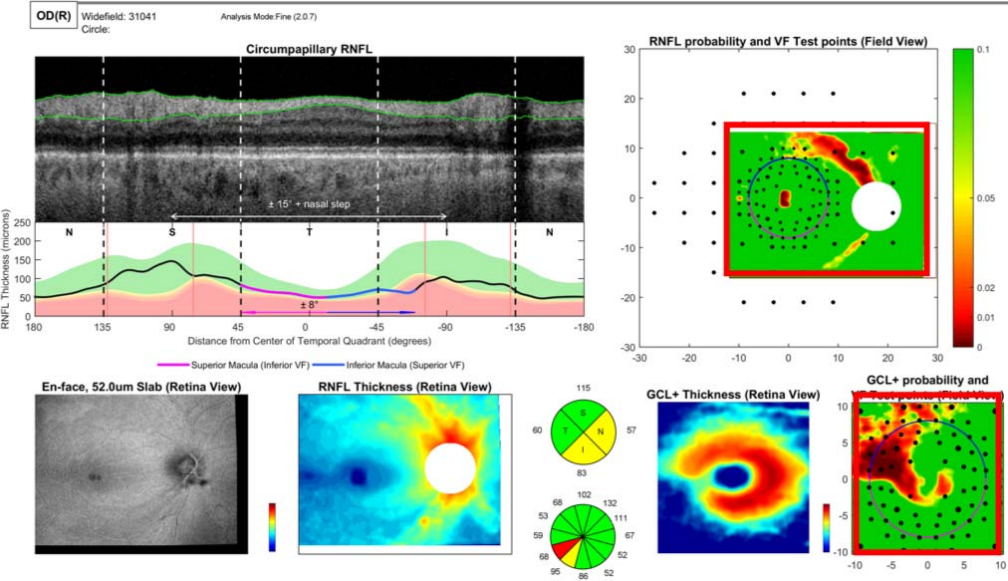
The report based upon a wide-field swept-source OCT volume scan (see text and Hood et al.^10^ and Wu et al.^11^). The RFNL (upper right, red rectangle) and RGC+ (lower right, red rectangle) probability maps are shown in field view (i.e., the top of each corresponds to the upper visual field/inferior retina).

For all eyes, wide-field volume scans were acquired by using a swept-source OCT instrument (Atlantis; Topcon, Inc., Tokyo, Japan). These scans were 12 × 9 mm and consisted of 256 B-scans, each with 512 A-scans. OCT wide-field reports were generated by using a reference database from the OCT device manufacturers (data provided by Topcon, Inc.), as previously described.^10^ This original report was modified so that all eyes were aligned on the same fovea-to-disc angle.^11^ This corrects for head-eye torsion, as well as perhaps some anatomical differences. Figure 1 shows a sample report. For the present study, the key aspects of this report are the two probability maps within the red rectangles, the lower one is for RGC plus inner plexiform layer (RGC+IPL) thickness within the macula and the upper one is for the RNFL thickness. These are deviation/probability maps, where the scale is continuous from dark red (<0.1%) to red (1%) to yellow (5%) to green (>10%). Both maps are in field view; that is, they are rotated along the horizontal meridian so the upper region corresponds to the superior VF and inferior retina. The symbols indicate the location of the 24-2 (larger symbols) and 10-2 (smaller symbols) test points.

### The Automated Topographic Method

**Figure 2:**
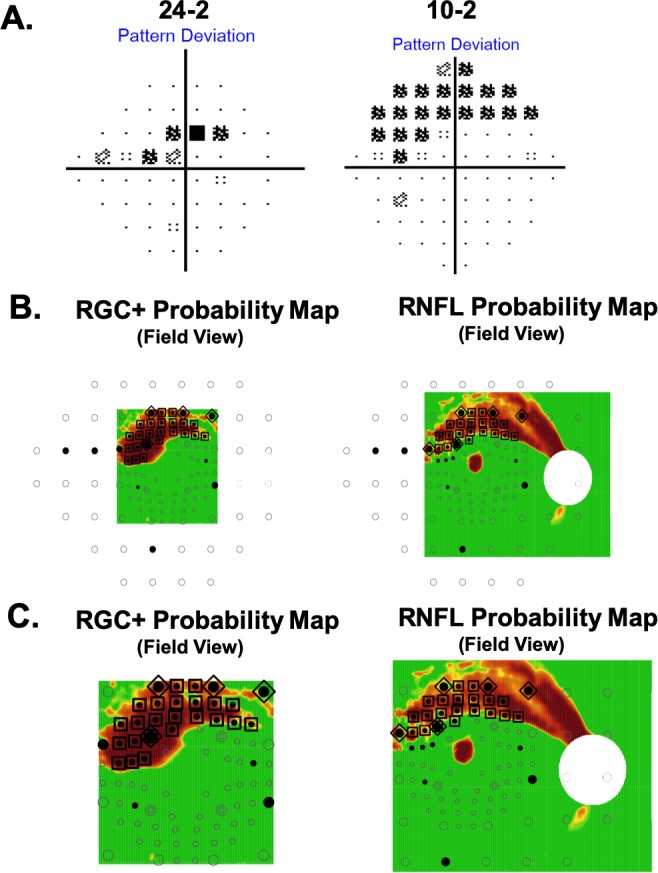
An eye with aS-aF agreement for both 24-2 and 10-2 VF locations on both RNFL and RGC+ layer probability maps. (A) The 24-2 and 10-2 VF for one of the eyes with early glaucoma. (B) RGC+ and RNFL probability plots shown with all the test locations of the 24-2 (larger circles) and 10-2 (smaller circles) VF. (C) An enlarged view of the portion of the maps in (B) covered by the OCT wide-field scan. The black filled circles are the VF locations significant at <5%. The probability scale for the OCT map varies continuously from green (P > 0.1) to dark red (P < 0.001). The locations enclosed in the large symbols indicate aS (<10%)–aF (<5%) agreement for the 24-2 (triangles) and 10-2 (squares) locations.

The topographic (T) agreement between the 24-2 and 10-2 VF deviation maps and OCT probability maps was objectively assessed by using a custom program developed in R.^12^ A complete description of this method can be found in a forthcoming publication (Tsamis et al., manuscript submitted, 2019). It is briefly described below and in Figure 2.

Figure 2A shows the 24-2 and 10-2 pattern deviation (PD) probability maps for one of the DG eyes. The output of the R-program is shown in Figure 2B. The OCT RGC plus inner plexiform layer (RGC+) and RNFL probability maps from Figure 1 are shown on the left and right side, respectively. The circles indicate the retinal locations of the 24-2 (large) and 10-2 (small) test spots. Note that the locations near fixation have been morphed to adjust for RGC displacement near the fovea, as previously described.^13^,^14^ The filled circles are the abnormal, *P* ≤ 0.05, VF points from panel A. The locations showing abnormal structure (aS) and abnormal function (aF) agreement are enclosed within large triangles (24-2) and squares (10-2). That is, for these locations, an abnormal VF point fell upon an OCT superpixel with *P* < 0.1. The superpixel had a diameter of a 1°, and was centered on the VF location.

For each eye, abnormal OCT structure–abnormal VF function (aS-aF) agreement was defined as two or more abnormal (≤5%) points on the combined 24-2 and 10-2 PD probability maps that fell upon abnormal (≤10%) OCT region on the RGC+ or RNFL probability maps. In Figure 2B, 26 locations (23 for 10-2 VF and 4 for 24-2 VF) showed aS-aF agreement on the RGC+ plot and 24 locations (20 for 10-2 VF and 5 for 24-2 VF) showed agreement on the RNFL plot. To make the results easier to see, Figure 2C shows enlarged images of the region common to the OCT and VF probability maps.

### A Metric Method: VF and OCT Summary Measures

For all eyes, the following summary measures were used for an M-method of defining aS-aF agreement.

#### Optical Coherence Tomography

To obtain the quadrant circumpapillary RNFL (cpRNFL) thickness measure, we used the wide-field, rotated OCT scan and derived a B-scan image for a circle 3.4 mm in diameter centered on the disc. For quadrant thickness, the average thickness of the cpRNFL layer was obtained for all eyes, including the normative database. The 5% and 1% limits were defined from the normative database and all DG and healthy control (H) eyes were coded as within normal limits (WNL) (green), ≤5% (yellow), or ≤1% (red). For quadrants, an eye was defined as “abnormal” as based upon quadrant cpRNFL if the superior (S), temporal (T), or inferior (I) quadrant was abnormal at the 5% (yellow) or 1% (red) level. We excluded the nasal (N) quadrant as it is outside the visual field. Note that including the N quadrant or excluding the T quadrant did not improve the performance.

#### Visual Fields

For the 24-2 VF, the summary measures included an MD ≤5%, a pattern standard deviation (PSD) ≤5%, a glaucoma hemifield test (GHT) “outside normal limits (ONL)”, and a combined PSD and GHT criterion of PSD ≤5% or GHT ONL.^15^ In addition, we combined the best of the 10-2 and 24-2 criteria. In particular, for a combined 24-2/10-2 definition, an eye was abnormal on VFs if it was abnormal on the 24-2 (PSD or GHT) or abnormal on the 10-2 (PSD).

## Results

### T-Method and Definite Glaucoma Eyes

All 53 eyes/patients in the (early) DG group and all 45 in the H group were included**.** These eyes were the primary focus of our analysis as they excluded suspects (PG), who may or may not have glaucomatous damage. For the 53 DG eyes, the best aS-aF agreement was obtained with the T-method when both 24-2 and 10-2 VFs were included. For the 53 DG eyes, there was 88.7% (47 eyes) agreement (Table 1, last column); only 6 eyes (11.3%) failed to show aS-aF agreement of abnormal regions. With only the 24-2 or only the 10-2, the aS-aF agreement fell to 64.2% and 77.4%, respectively, as shown in Table 1.

**Table 1:** A Comparison of aS-aF Agreement for 24-2 and/or 10-2 Visual Fields

	**T- Method aS-aF Agreement**		
	**24-2**	**10-2**	**Combined 24-2 and 10-2**
53 DG eyes	34 (64.2%)	41 (77.4%)	47 (88.7%)
45 H eyes	2 (4.4%)	1 (2.2%)	2 (4.4%)

#### Need for Both 24-2 and 10-2 VFs

**Figure 3:**
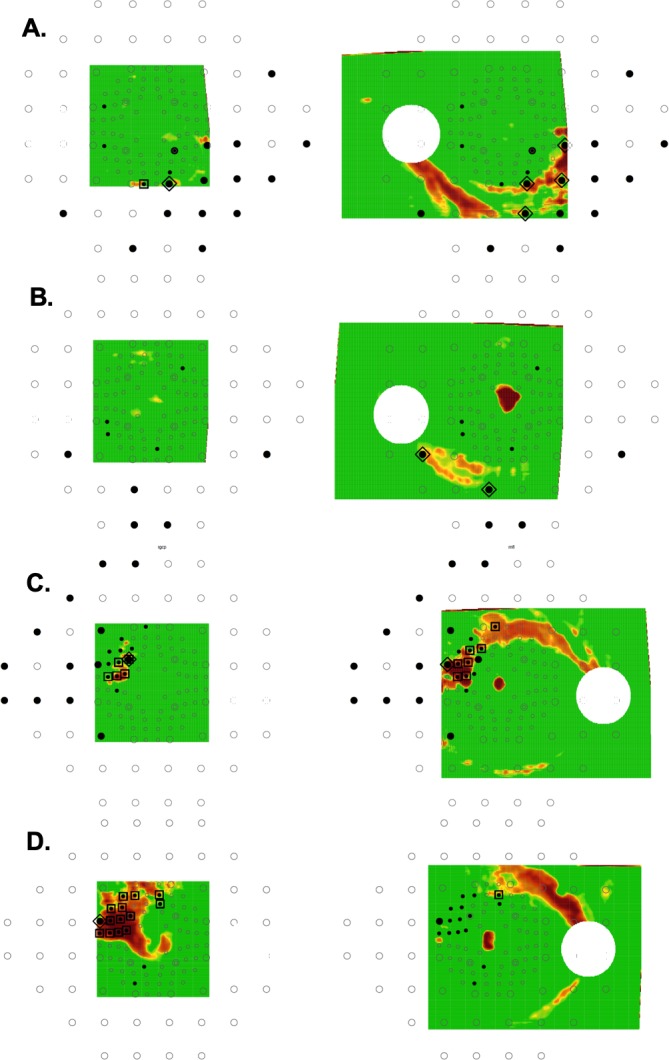
RGC+ (left) and RNFL (right) probability maps with superimposed VF location as in Figure 2B. (A, B) Two of the four eyes with aS-aF agreement for 24-2 VF, but not for 10-2 VF. (C, D) Two of the 11 eyes with aS-aF agreement for the 10-2 VF, but not for the 24-2 VF.

Of the 47 of 53 DG eyes showing aS-aF agreement with the combined 24-2 and 10-2 locations, 29 eyes showed agreement for either 10-2 or 24-2 locations, while 12 showed agreement based upon the 10-2 VF locations, but not the 24-2 VF locations, and 5 showed agreement for the 24-2 VF locations, but not the 10-2 VF locations; while 1 eye required locations from both 24-2 and 10-2 to show agreement. Figure 2 is an example of 1 of the 29 eyes showing aS-aF agreement with either the 10-2 VF (large squares), or the 24-2 VF (large triangles). Figures 3A and 3B show the results for two of the five eyes with aS-AF agreement for 24-2 VF (large triangles), but not for 10-2 VF. All five had OCT arcuate abnormalities that were largely outside the macula, as expected. In three cases, (e.g., Fig. 3A), these OCT arcuate abnormalities were clear and extensive, while in the other two cases, (e.g., Fig. 3B), the OCT abnormalities were relatively subtle. Figures 3C and 3D show the results for 2 of the 12 eyes with agreement for the 10-2 VF (large squares), but not for the 24-2 VF.

#### Need for Both RGC+ and RNFL Probability Maps

Of the 47 eyes showing aS-aF agreement, 35 showed agreement for both the RGC+ and RNFL maps, while 7 showed agreement for the RGC+ map, but not the RNFL map (e.g., Fig. 3D); and 5 showed agreement for the RNFL map, but not the RGC+ map (e.g., Fig. 3B).

#### DG Eyes Without aS-aF Agreement

Six (11.3%) of the 53 eyes failed to show aS-aF agreement in abnormal regions. Note that these are not false negatives (FNs) as we are not suggesting that aS-aF agreement be used to define glaucoma (see Discussion).

**Figure 4:**
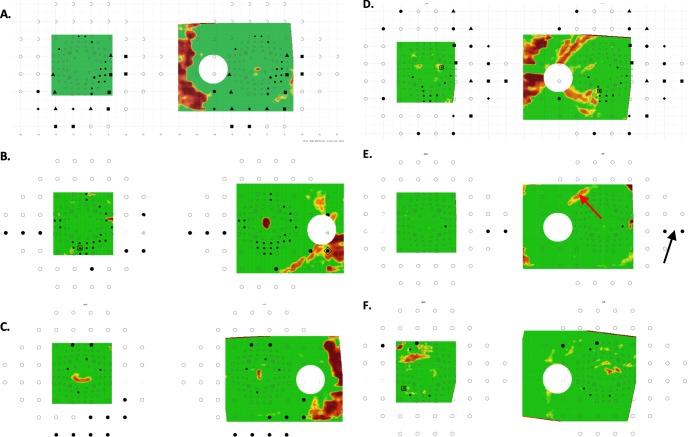
Same as in Figure 3 for the six DG eyes that failed to show aS-aF agreement. (A–C) Three eyes with abnormal arcuate regions that were more obvious on the 24-2 VFs (A–C), and 10-2 VFs (A, B) probability maps than they were on the OCT probability maps. (D) An eye with an arcuate defect seen on 24-2 and on the OCT RNFL plot; however, the abnormal VF and OCT regions did not spatially overlap. (E, F) Two eyes with abnormal regions on both OCT and VFs that are subtle and/or difficult to discern.

In three of the six eyes (Figs. 4A–C), the abnormal arcuate regions were more obvious on the VFs than they were on the OCT. In these three eyes, the defects were seen in the same region on three repeated VFs obtained on subsequent test days. A fourth eye (Fig. 4D) also had arcuate defects seen on 24-2, and these also replicated on 3 other test days. In this case, the corresponding damage is clear on the OCT RNFL plot. However, the abnormal VF and OCT regions did not spatially overlap. For the remaining two eyes (Figs. 4E, 4F), the abnormal regions on both OCT and VFs are subtle and/or difficult to discern. In the case of Figure 4E, there appears to be a nasal step in the inferior 24-2 VF (black arrow) and a hint of arcuate abnormality on the RNFL plot (red arrow) in the corresponding location. It is important to note that in all six cases, there was little evidence of structural (OCT) damage preceding functional (VF) damage, while one could argue the reverse in the case of the three eyes in Figures 4A through 4C.

#### Healthy Eyes With aS-aF Agreement

**Figure 5:**
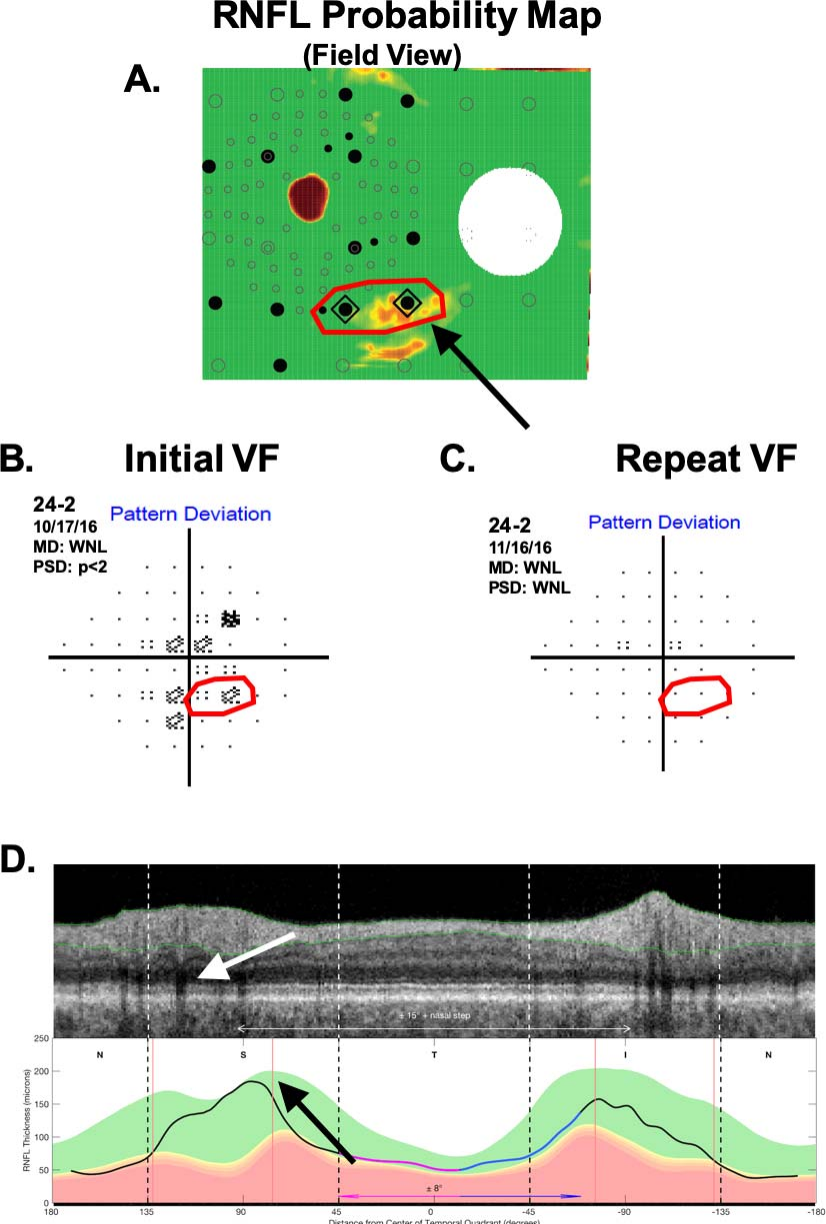
One of the two H eyes showing aS-aF agreement. (A) The RNFL probability plot. (B) The associated 24-2 pattern deviation plot. (C) A 24-2 pattern deviation plot for a later visit. (D) The derived OCT circumpapilllary image and cpRNFL thickness plot.

**Figure 6:**
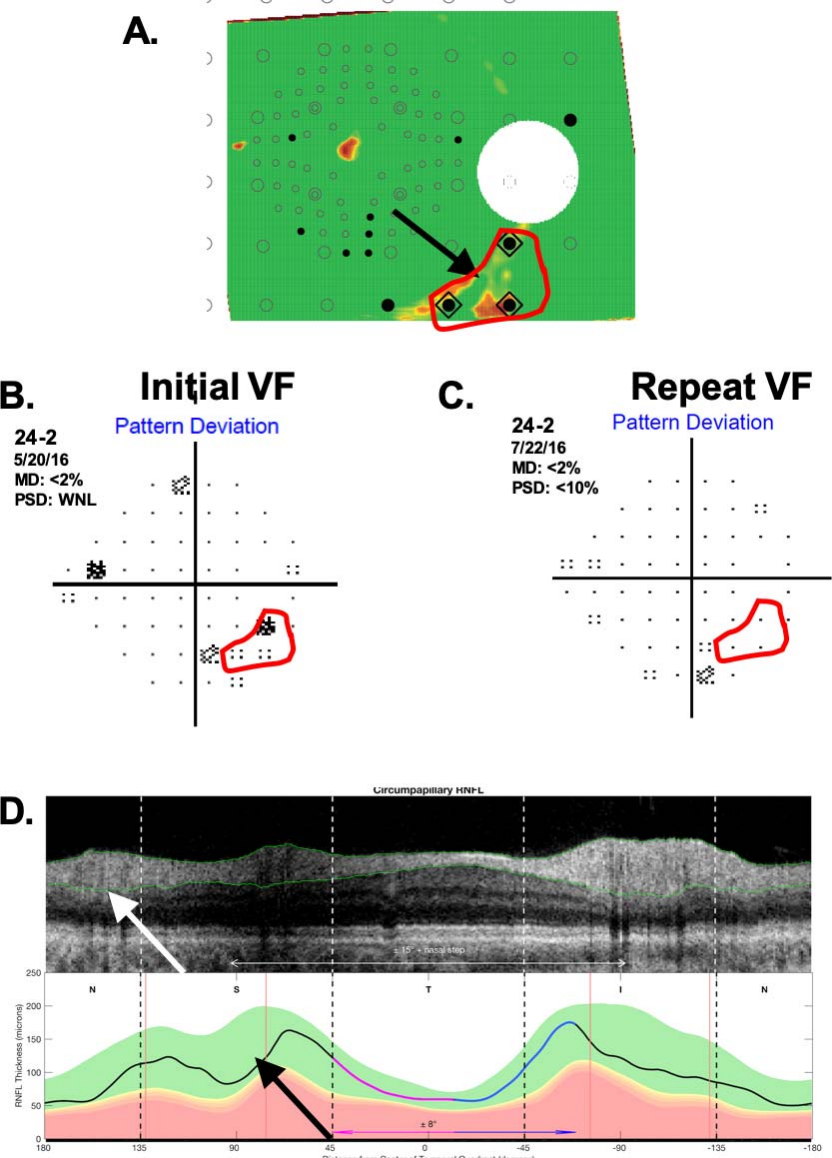
Same as Figure 5 for the other H eye showing aS-aF agreement.

Only 2 (4.4%) of the 45 healthy eyes showed abnormal aS-aF agreement. Figures 5 and 6 show the RNFL probability plots (panel A) for these eyes with the relevant aS-aF points from the 24-2 VF (panel B) shown within the red boundaries. Notice that these locations are not abnormal in the repeated 24-2 VFs (panel C). Further, these very subtle arcuate-like abnormal regions on the RNFL probability plot (black arrows) can be identified as artifacts if the derived circumpapilllary image (upper panel in Figs. 5D and 6D) and the cpRNFL thickness plots (lower panel D) are examined. In both cases, the cpRNFL thickness is in the normal (green) regions, and the scans suggest the reason for the artifactual arcuates in panel A. In Figure 5D, the location of the superior temporal blood vessels (white arrow) deviates from the average location (red line, black arrow). In Figure 6D, there is an unusually large “split bundle” (white arrow).

### An M-Method for Abnormal Structure-Abnormal Function Agreement

#### VF Summary Measures

To obtain an M-method to compare to our T-method, we first need to choose among the available 24-2 and 10-2 summary measures. Table 2 provides a summary of the DG eyes missed (i.e., false positive [FP]) and H eyes mistakenly classified as abnormal (FN) for the common VF measures. Among the 24-2 measures, the “PSD OR GHT” criterion had the fewest FNs, 15, and the lowest total number, 21, of “mistakes” (FN+FP). For the 10-2 VF, the PSD did better than the MD.

**Table 2:** A Comparison of Alternative Definitions of Abnormal Visual Fields

	**24-2**				**10-2**		**Combined**
	**24-2 MD**	**24-2 PSD**	**24-2 GHT**	**24-2 PSD OR GHT**	**10-2 MD**	**10-2 PSD**	**24-2 PSD OR GHT OR 10-2 PSD**
False negative, *N* = 53 DG	29 (54.7%)	17 (32.1%)	22 (41.5%)	15 (28.3%)	31 (58.5%)	26 (49.1%)	11 (20.8%)
False positive, *N* = 45 healthy	12 (26.7%)	6 (13.3%)	3 (6.7%)	6 (13.3%)	10 (22.2%)	2 (4.4%)	6 (13.3%)
Total (FN+FP), *N* = 98	41 (41.8%)	23 (23.5%)	25 (25.5%)	21 (21.4%)	41 (41.8%)	28 (28.6%)	17 (17.3%)

The combination of 24-2 and 10-2 measures had the fewest FNs, 11, and the lowest total number, 18, of mistakes (last column of Table 2). Recall that for this combination, an eye was classified as abnormal if either the 24-2 GHT OR 24-2 PSD OR 10-2 PSD value was abnormal at 5% or less. In any case, while the combined 24-2 and 10-2 performed the best, it still missed (FN) 11 (20.8%) of the eyes classified as DG and falsely classified as abnormal (FP) 6 (13.3%) of the healthy eyes.

#### OCT Summary Measures

The quadrant (Q-yellow or red) OCT metric had the best performance with 8 (15.1%) FNs and only 1 FP (2.2%). It did better than any other global or sector method we tried. For example, a Q-red metric based upon <1% (red) rather than <5% (yellow) showed 23 (43.4%) FNs. It also did better than any of the VF measures in Table 2.

#### A Metric Method

**Figure 7:**
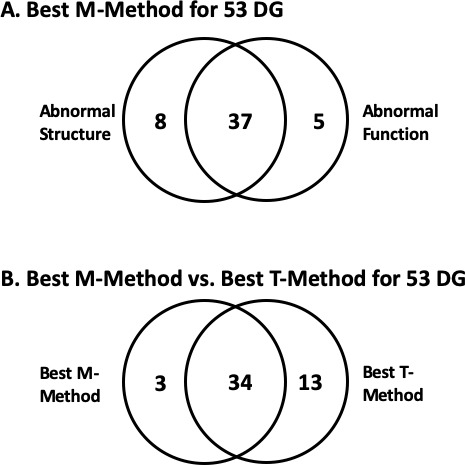
Venn diagrams summarizing the number of 53 DG eyes showing (A) aS and/or aF for the M-method and (B) aS-aF agreement based upon the best T- and M-methods.

For the M-method, we combined the OCT Q criterion with the best 24-2 alone (GHT OR PSD) and best combined 10-2 (PSD) OR 24-2 (GHT OR PSD). Table 3 shows the results expressed as aS-aF agreement as in Table 2. When using only 24-2 VF (GHT OR PSD) combined with OCT Q criterion, 20 (37.7%) of the 53 DG eyes failed to show aS-aF agreement. By combining 24-2 and 10-2, aS-aF agreement improved by four eyes, with 37 (69.8%) of the 53 DG eyes showing agreement (Fig. 7A). However, 16 eyes (30.2%) still failed to show agreement, compared to 6 (11.3%) for the T-method.

**Table 3:** A Comparison of aS-aF Agreement for 24-2 and/or 10-2 Visual Fields

	**M-Method aS-aF Agreement**	
	**24-2 PSD OR GHT AND Q**	**24-2 PSD OR GHT OR 10-2 PSD AND Q**
53 DG eyes	33 (62.3%)	37 (69.8%)
45 H eyes	0 (0%)	0 (0%)

In sum, for the best T-method, 47 (88.7%) of the 53 DG eyes showed aS-aF agreement, while for the best M-method it was 37 (69.8%), with 34 of these eyes showing agreement with both T- and M-methods (see Fig. 7B.) Note that in both cases the best method required the 10-2, as well as the 24-2 VF.

### The Probably Glaucoma Eyes

An additional 17 eyes were classified as PG. Adding these eyes to the analysis decreased the percentage of eyes showing aS-aF, but did not change the conclusion concerning M- versus T-methods. In particular, of the 17 PG eyes, 4 (23.5%) and 11 (64.7%) showed aS-aF agreement for the M- and T-methods, respectively.

## Discussion

We have previously argued that, in general, the extent to which structural and functional measures of glaucomatous damage agree, as well as in particular which appears to come first, will depend upon the particular tests performed and how these tests are analyzed.^5^,^7^ Here we used an automated topographic approach and compared the abnormal regions on 24-2 and 10-2 PD deviation/probability maps to those on OCT RGC+ and RNFL probability plots (Tsamis et al., manuscript submitted, 2019). Such an automated approach overcomes the subjective nature of our previous work.^7^,^16^ Because various approaches may produce good agreement in severe, and even moderate, glaucoma, we studied eyes classified as early glaucoma, based upon a 24-2 MD better than −6 dB. To avoid including eyes with an uncertain diagnosis, we restricted the study to eyes classified by the referring physician as “definite glaucoma” (DG) and to healthy eyes recruited with a normal IOP and fundus exam. There are several aspects of the results worth discussing.

### Abnormal Regions on VF and OCT Show Good Agreement in Most Eyes

In general, with the fully automated T-method, structural and functional damage due to early glaucoma showed good agreement in most eyes. Almost 90% of the DG eyes showed topographic agreement of abnormal regions. While two healthy eyes also showed aS-aF agreement with our T-method, they were easily identified as mistakes. First, the abnormal aS-aF agreement did not replicate on repeated VFs. Second, both eyes had an arcuate-like artifact on the RNFL probability plot, which an experienced OCT reader would identify as being due to normal anatomical variations (Figs. 6, 7).

Although the M-method that combined 24-2 and 10-2 VF and OCT summary measures did not do as well, it also showed agreement in most (70%) of DG eyes. However, over 2.5 times as many of the 53 DG eyes failed to show aS-aF with the M-method (16 eyes), as compared with our T-method (6 eyes). With only the 24-2 VF, 20 (37.7%) of the 53 DG eyes failed to show aS-aF agreement with the M-method.

### The Need for a 10-2 Type Pattern

The evidence is clear that early glaucomatous damage often, in fact typically, includes the macular region, defined as ±8° from fixation (see summaries in Hood^16^ and Hood et al.^17^). Further, this damage can be missed with the 24-2 VF because the 24-2 pattern has only 4 test points in this region.^18^ Thus, it is not surprising that our T-method did better with the combined 24-2 and 10-2 VFs (89% agreement for DG eyes) than it did with only the 24-2 VF (64% agreement for DG eyes).

Recently, the 24-2c VF pattern Humphrey Field Analyzer (Carl Zeiss Meditec, Inc.) has been proposed as an alternative to performing separate 24-2 and 10-2 VFs, which has logistic problems in clinical practice. The 24-2c pattern includes the conventional 24-2 locations enhanced with 10 locations from the 10-2, five in the inferior and five in the superior field, along the lines suggested by Ehrlich et al.^19^ As expected, more DG eyes showed aS-aF agreement with the 24-2c (40 eyes) than the 24-2 alone (34 eyes), but fewer than the number of eyes (47) with the combined 24-2 and 10-2 locations. In other words, the 24-2c “missed” seven of the eyes showing aS-aF agreement with the combined 24-2 and 10-2 VFs.

### The Need for RGC+ and RNFL Probability Maps

As we have previously argued,^18^ RGC+ probability maps can detect damage missed by RNFL probability maps, and vice versa. Thus, both are needed for clinical judgments. The same argument applies to the T-method in the present study. Of the 47 eyes showing aS-aF agreement, seven fewer would have shown agreement without the RGC+ plot, and four fewer would have shown agreement without the RNFL plot.

### Which Comes First, Structural (OCT) or Functional (VF) Damage?

In general, our data are consistent with those studies that argue that to a first approximation, the degree of structural (i.e., local cpRNFL thickness) and functional (VF local sensitivity) loss agree.^5^–^9^ However, the answer to the question “Which comes first, structural (OCT) or functional (VF) damage?” is more nuanced. First, to answer this question, one needs to specify both the particular structural and functional tests performed, as well as the measures used for comparison. For example, if for our M-method we had used the 24-2 test and MD as our VF measure and an abnormal quadrant as our OCT measure, then less than one-half of the 53 DG eyes would have shown aS-aF agreement. Second, this does not mean that the degree of structural or functional changes will always agree for a particular eye. Whether structural or functional damage appears to come first will depend upon the baseline values of each measure for that eye as explained in Hood and Kardon,^5^ as well as the within and between individual variability of the measures.

### The T-Method and Definitions of Glaucoma

The National Eye Institute's (NEI's) statement on NEEDS regarding diagnosis, imaging, and biomarkers states that we need to “establish a consensus definition of POAG and standardize tools to assess its various phenotypes.”^20^ While our automated method, which includes both 10-2 and 24-2 VFs, overcomes one problem we see in defining POAG (i.e., aS-aF agreement), there are others worth mentioning. First, many of the databases available for testing definitions of glaucoma do not have 10-2 VF and/or OCT scans that include the macula. Second, in some cases, these databases have only summary measures, and not the pattern of results (e.g., VF total or pattern deviation maps) needed for a T-method. Third, as there is no litmus test for POAG, there is no one accepted reference standard to test alternative POAG definitions. If we use the judgment of glaucoma experts, this raises a host of other concerns, such as the information they use and how they use it. This does not mean we cannot take steps to address the NEI concern that “Absence of a highly sensitive and specific definition of POAG remains a major impediment to sharing data and gaining further insights into genes, risk factors, diagnosis, and treatment.”^20^ We can move toward the NEI goal by agreeing on the minimum information (e.g., 24-2 VF GHT, MD, PSD or OCT global or quadrant thickness) a research paper should include regarding its patient population. However, it is probably not time for a single quantitative definition of POAG.

### Limitations

There were several limitations of this study worth discussing. First, the wide-field OCT scan covers a smaller region of the retina than does the 24-2 VF. On the other hand, even if it were possible to extend the region covered by the OCT so that it included, for example, the region containing the 24-2 nasal step defect, it is unlikely to have a major effect on the aS-aF comparison, as the OCT RNFL becomes relatively thin near the raphe, and the RGC+ layer, relatively thin outside the macula. On the other hand, there was a second, and related, limitation that can also lead to an underestimate of the extent of aS-aF agreement. In particular, by relying on pointwise agreement between regions of aS and aF, we may be underestimating the degree of aS-aF agreement. Consider Figure 4D (right panel). There is clear aS-aF agreement missed by our T-method, but apparent if one takes into consideration the predicted projection of the RNFL damage suggested by the OCT RNFL map. To deal with both limitations, we are exploring ways to define the extent of the arcuate damage. The arcuate region of damage can be estimated from tracings of RNFL bundles^21^ (see figures 17 and 18 in Hood^16^; figure 6 in Tsamis et al., manuscript submitted, 2019). Alternatively, a model customized for individual eyes^22^ might be useful in describing the arcuate regions associated with regions of damage at the disc. This same model can be used to adjust the maps, based upon anatomical variations across eyes. However, the improvement in agreement over and above the adjustment we make for fovea-to-disc angle in the present study warrants further investigation.^23^

A third limitation affecting nearly all aS-aF studies is the lack of a gold standard for glaucomatous damage. Because we are interested in aS-aF agreement in eyes that have glaucoma, we focused on eyes classified as “definite glaucoma” by the referring specialist. While it could be argued that specialists are using “good structure-function agreement” in arriving at their “definite” diagnosis, it does not follow that this would favor a T-method as opposed to the M-method. In fact, the latter uses the summary metrics (e.g., PSD and GHT) often favored by clinicians. In any case, the PG (probably glaucoma) data also agree with our conclusion that the agreement between aS and aF is better with our T-method than with the typical M-methods.

Finally, the fact that the T-method was optimized on the same sample (i.e., 53 DG eyes and 45 H eyes) (Tsamis et al., manuscript submitted, 2019) may overestimate its performance. However, we previously have validated this method on an independent data set (Tsamis et al., manuscript submitted, 2019) where it did as well or even better. Further, we did attempt to achieve the best sensitivity/specificity for the M-method on the same database as well. In addition, the application of T- and M-methods on the PG eyes, which is a sample unknown to both methods, revealed similar results.

## Conclusions

Using an automated and objective method, we found that if OCT and VF deviation/probability maps are compared, then structural and functional damage due to confirmed glaucoma shows good agreement in most eyes, even those with relatively early damage. However, these comparisons depend upon using both 10-2 and 24-2 VFs, and OCT scans that allow for both RGC+ and RNFL analyses. Finally, our findings do not support the general premise that structure precedes function in glaucoma. The extent to which this is true depends upon the VF and OCT tests performed, the measures of agreement used, as well as the VF sensitivity and OCT RGC+ and RNFL thickness when healthy.
